# A nomogram for predicting cerebral white matter lesions in elderly men

**DOI:** 10.3389/fneur.2024.1343654

**Published:** 2024-05-01

**Authors:** Yi-Feng Pei, Xian-Dong Li, Quan-Ying Liu, Chu-Wei Zhang, Yi-Han Wang, Ming-Rui Chen, Hui-Sheng Chen

**Affiliations:** Department of Neurology, General Hospital of Northern Theater Command, Shenyang, China

**Keywords:** cerebral white matter lesions, prediction model, nomogram, elderly, male

## Abstract

**Objective:**

This study aimed to develop a nomogram tool to predict cerebral white matter lesions (WMLs) in elderly men.

**Methods:**

Based on a retrospective cohort from January 2017 to December 2019, a multivariate logistic analysis was performed to construct a nomogram for predicting WMLs. The nomogram was further validated using a follow-up cohort between January 2020 and December 2022. The calibration curve, receiver operating characteristics (ROC) curves, and the decision curves analysis (DCA) were used to evaluate discrimination and calibration of this nomogram.

**Result:**

A total of 436 male patients were enrolled in this study, and all 436 patients were used as the training cohort and 163 follow-up patients as the validation cohort. A multivariate logistic analysis showed that age, cystatin C, uric acid, total cholesterol, platelet, and the use of antiplatelet drugs were independently associated with WMLs. Based on these variables, a nomogram was developed. The nomogram displayed excellent predictive power with the area under the ROC curve of 0.951 [95% confidence interval (CI), 0.929–0.972] in the training cohort and 0.915 (95% CI, 0.864–0.966) in the validation cohort. The calibration of the nomogram was also good, as indicated by the Hosmer–Lemeshow test with *p*-value of 0.594 in the training cohort and 0.178 in the validation cohort. The DCA showed that the nomogram holds good clinical application value.

**Conclusion:**

We have developed and validated a novel nomogram tool for identifying elderly men at high risk of WMLs, which exhibits excellent predictive power, discrimination, and calibration.

## Introduction

Cerebral white matter lesions (WMLs) are not only an important part of cerebral small vessel disease (SCVD), but also one of the most common causes of cognitive impairment in the elderly, and considered to be an early sign of brain damage (1, 2). The incidence of WMLs in the elderly has been reported to range from 11% to 21%, with rates reaching as high as 94% in advanced-aged individuals (3). Given the increasing global aging population, research on WMLs has gained significant attention (4). Growing evidence showed that heavier WMLs burden was closely associated with more severe cognitive impairment (5, 6). Some studies have shown that the location of WMLs was closely related to the prognosis of patients with acute ischemic stroke (7), and the severity of WMLs may affect the functional prognosis of ischemic stroke (8). Other studies suggest that WMLs can increase the risk of dementia and death in stroke patients (3). Furthermore, a prospective study revealed that severe WMLs can predict composite endpoints of death, pneumonia, and falls (9).

Some studies have identified the correlations of age, hypertension, female gender, high density lipoprotein (HDL) levels, and the use of antihypertensive medications with occurrence of WMLs (10, 11). However, accurately predicting the occurrence of WMLs remains a challenge. Only one recent study reported a prediction model focused on the elderly (10). However, constructing models that incorporate a wider range of clinically relevant variables could help enhance the accuracy of WMLs prediction, which is crucial for identifying the appropriate population for early intervention.

In this context, the current study aims to develop a predictive nomogram based on comprehensive baseline characteristics of elderly men.

## Methods

### Patients and study design

We retrospectively enrolled consecutive patients who underwent comprehensive medical examination including brain magnetic resonance imaging (MRI) between January 2017 and December 2019. Eligible patients were used as a training cohort to develop a nomogram for predicting WMLs. After this time, all these patients were retrospectively followed up for 3 years (between January 2020 and December 2022) as the validation cohort. Patients fulfilling the following eligibility criteria were included for analysis: (a) age ≥ 60 years; (b) complete brain MRI including T2-weighted images and fluid-attenuated inversion recovery (FLAIR); (c) complete medical examination. The exclusion criteria: (a) incomplete clinical data; (b) large area cerebral infarction, cerebral hemorrhage, subarachnoid hemorrhage and craniocerebral trauma; (c) severe liver dysfunction, severe cardiac dysfunction, hypothyroidism, gout, autoimmune system diseases, severe systemic infection; (d) taking immunosuppressant recently; (e) taking B vitamins and folic acid recently; (f) taking uric acid-lowering drugs recently; (g) other conditions known to cause WMLs, such as carbon monoxide poisoning, hypoxic encephalopathy, various types of hydrocephalus, immune white matter demyelination and so on; (h) diseases that may interfere with the analysis of WMLs, such as brain tumors, cysts and history of craniocerebral surgery, etc. This study was approved by the Ethics Committee of General Hospital of Northern Theater Command, Shenyang and waived the need for informed consent from all subjects.

### Data collection

The following demographic and clinical characteristics were recorded: age, weight, smoking and drinking history, admission systolic blood pressure (SBP) and admission diastolic blood pressure (DBP), medical comorbidity [hypertension, diabetes, coronary heart disease (CHD), atrial fibrillation (AF), left heart failure (LHF) and tumor]; the use of statins, antiplatelet drugs and anticoagulants. Laboratory test values including serum total cholesterol (TC), triglyceride (TG), high density lipoprotein (HDL), low density lipoprotein (LDL), lipoprotein-A (LP-A), albumin (ALB), total protein (TP), high-sensitivity C-reactive protein (h-CRP), platelets (PLT), glycosylated hemoglobin (HbA1C), total bilirubin (TBIL), direct bilirubin (DBIL), indirect bilirubin (IBIL), alanine aminotransferase (ALT), gamma glutamyl transpeptidase (GGT), homocysteine (Hcy), cystatin C (Cys C), uric acid (UA), creatinine (CREA), urea nitrogen (UREA) and brain natriuretic peptide (BNP).

### WMLs assessment

The WMLs were evaluated utilizing the Fazekas scale on MRI (12), and were defined as any occurrence of WMLs (namely a Fazekas score of at least 1 point). In the scale, periventricular and deep WMLs were rated separately, and periventricular WMLs were graded according to the following patterns: 0 = absent; 1 = caps or pencil-thin lining; 2 = smooth halo; and 3 = irregular periventricular WMLs extending into a deep WMLs. Deep WMLs were graded according to the following patterns: 0 = absent; 1 = punctate foci; 2 = beginning confluence of foci; and 3 = large fused areas. A total Fazekas score, ranging from 0 to 6, was acquired by summing the periventricular and deep WMLs scores. Based on the Fazekas scale, WMLs did not include old lacunar infarctions and/or white matter lesions at some other locations such as basal ganglia, deep gray matter and cortical/subcortical areas. The neuroimaging data were evaluated independently by two experienced neurologists who were blinded to the clinical data. Any disagreement between the two assessors was resolved by discussion until a consensus was achieved.

### Statistical analysis

The patient data in the training cohort were used to develop the prediction model, and the patient data in the validation cohort were used to validate the model. Gaussian distributed data were expressed as means ± standard deviation (SD) and compared by student’s t-test, while non-Gaussian distributed data were expressed as medians (interquartile range, Q2–Q3) and compared by the Mann–Whitney U test. Categorical data are expressed using frequencies and ratios (%) and Fisher’s exact tests or the χ2 tests were used for categorical variables. SPSS 26.0 was used for statistical analyses. All variables with a probability value < 0.10 in the univariate analysis entered into a multivariate logistic regression analysis using a backwards stepwise method. Stata software (version 17.0) was used to build the nomogram prediction model. The nomogram converts each independent risk factor included in the model into an assessment point system. The total points obtained determine the final risk assessment value. The odds ratio (OR) and 95% confidence interval (CI) of each significant risk factor in the final logistic regression model were calculated. Performances of the nomogram were assessed in the validation cohort. Discrimination of the nomogram was assessed using the area under the ROC curve (AUC). An AUC of 0.51–0.7 indicates low accuracy, 0.71–0.9 indicates moderate accuracy, and 0.91–1.0 indicates high accuracy to discriminate between patients with WMLs and without WMLs. The calibration of the nomogram was evaluated by the Hosmer-Lemeshow test (*p* > 0.05 indicates good calibration). In addition, a decision curve analysis (DCA) of the model was developed to quantify the net benefit rate at different threshold probabilities to assess the clinical validity of the model. All statistical tests were 2-tailed. We deemed statistical significance at *p* = 0.05.

## Results

### Demographic and clinical features

**Figure 1:**
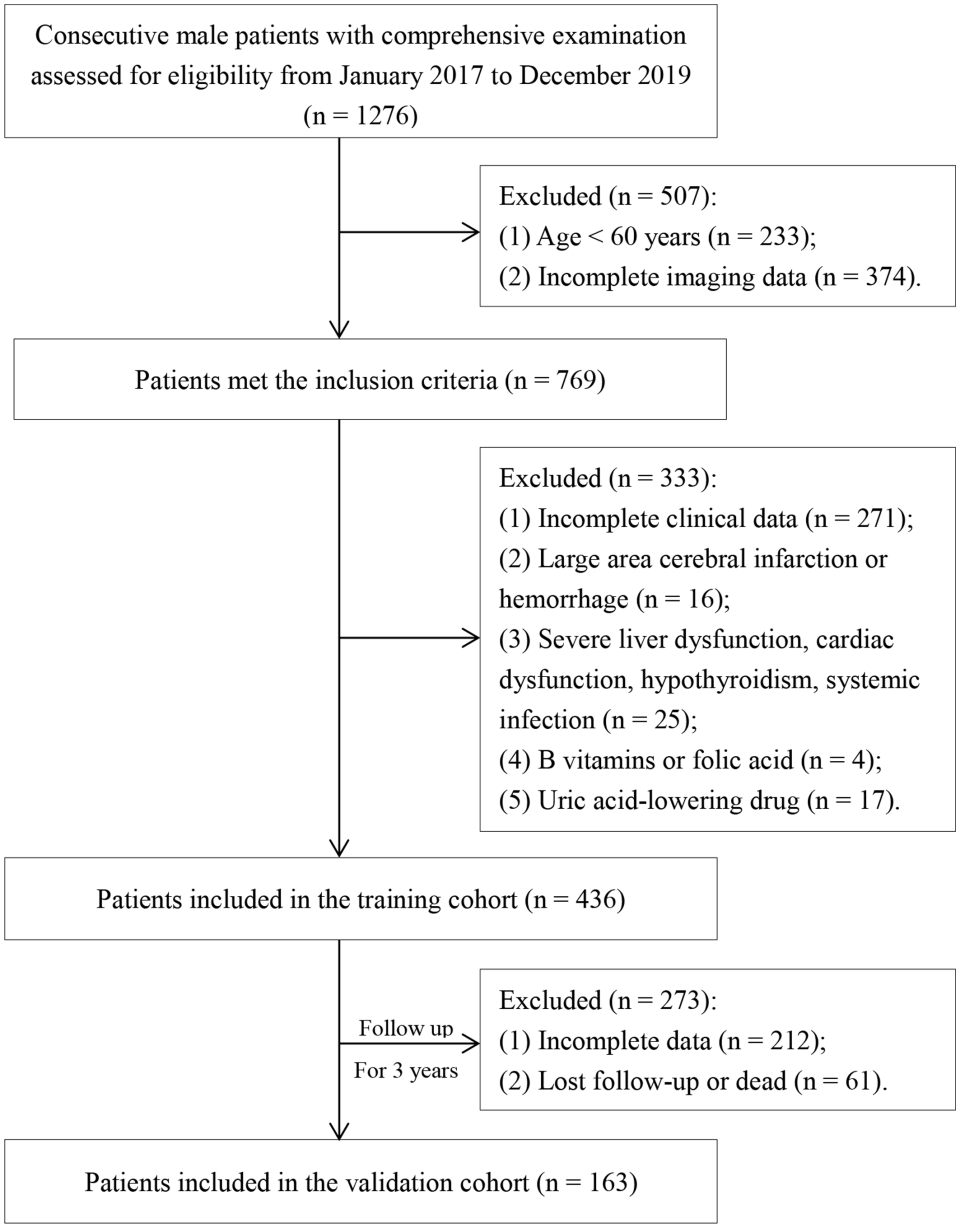
Flowchart of participant recruitment.

As shown in Figure 1, 1,276 patients were screened for eligibility and 436 patients were included in the training cohort after excluding 840 individuals. The patients in the training cohort were followed for 3 years. However, due to incomplete follow-up information, 273 patients were subsequently excluded. As a result, a total of 163 follow-up patients were included in the validation cohort. The demographics and clinical characteristics of the patients in the two cohorts were shown in Table 1. There was a good balance in the baseline characteristics between the training and validation cohort except for tumor history, statins history, uric acid and serum total protein. The average age of patients in the training cohort and validation cohort were 73.93 ± 14.15 and 75.22 ± 11.84 years old, respectively. The prevalence rate of WMLs in the training cohort was 65.6%, whereas in the validation cohort was as high as 82.6%.

**Table 1:** Demographic and clinical features of the participants.

Variables	Training cohort (*n* = 436)	Validation cohort (*n* = 163)	*p*
Age (year)	73.93 ± 14.15	75.22 ± 11.84	0.243
Weight (kg)	71.69 ± 9.75	71.84 ± 9.48	0.867
Coronary heart disease (%)	194 (44.5)	73 (44.8)	0.949
Hypertension (%)	290 (66.5)	108 (66.3)	0.953
Diabetes mellitus (%)	143 (32.8)	60 (36.8)	0.356
Atrial fibrillation (%)	46 (10.6)	19 (11.7)	0.699
Tumor (%)	97 (22.2)	49 (30.1)	0.047
Left heart failure (%)	40 (9.2)	17 (10.4)	0.461
Smoking (%)	146 (33.5)	52 (31.9)	0.714
Drinking (%)	173 (39.7)	65 (39.9)	0.965
Statins (%)	315 (72.2)	135 (82.8)	0.008
Antiplatelet drugs, *n* (%)	151 (34.6)	49 (30.1)	0.291
Anticoagulants, *n* (%)	41 (9.4)	15 (9.2)	0.940
Systolic blood pressure (mmHg)	133.98 ± 13.34	133.85 ± 15.19	0.919
Diastolic blood pressure (mmHg)	74.73 ± 9.35	73.94 ± 9.19	0.357
Homocysteine (umol /L)	12.04 ± 4.49	12.08 ± 3.57	0.926
Cystatin C (mg/L)	1.09 ± 0.66	1.15 ± 0.42	0.346
Uric acid (umol /L)	345.76 ± 75.59	366.80 ± 74.58	0.002
Creatinine (mmol/L)	81.01 ± 57.80	80.64 ± 19.84	0.937
Urea nitrogen (mmol/L)	6.09 ± 2.45	6.37 ± 1.91	0.188
h-CRP (mg/L)	1.82 ± 2.19	1.99 ± 2.97	0.457
Total bilirubin (umol /L)	11.29 ± 4.75	11.50 ± 5.01	0.629
Direct bilirubin (umol /L)	3.62 ± 1.85	3.34 ± 3.44	0.193
Alanine aminotransferase (U/L)	20.68 ± 11.56	20.33 ± 9.36	0.729
GGT (U/L)	29.02 ± 17.70	30.31 ± 39.25	0.579
Total cholesterol (mmol/L)	4.07 ± 0.95	4.05 ± 1.01	0.826
Triglyceride (mmol/L)	1.45 ± 1.14	1.46 ± 0.78	0.861
High density lipoprotein (mmol/L)	1.17 ± 0.29	1.13 ± 0.31	0.181
Low density lipoprotein (mmol/L)	2.23 ± 0.68	2.18 ± 0.73	0.415
Lipoprotein-A (g/L)	209.20 ± 196.70	212.32 ± 213.73	0.866
Total protein (g/L)	66.67 ± 5.55	65.25 ± 5.33	0.005
Albumin (g/L)	39.88 ± 15.49	38.39 ± 4.11	0.226
Brain natriuretic peptide (pg/mL)	208.16 ± 387.89	226.01 ± 443.10	0.630
Glycosylated hemoglobin (%)	5.96 ± 0.75	5.99 ± 0.85	0.562
Platelet (×10^9^/L)	202.38 ± 65.24	211.23 ± 76.18	0.159
Cerebral white matter lesions (%)	286 (65.6)	134 (82.2)	<0.001

Values are mean SD or *n* (%). h-CRP, high-sensitivity C-reactive protein; GGT, gamma glutamyl transpeptidase. Numerical variables were analyzed with Student’s t-test. For categorical variables, chi-square or Fisher exact test was used.

### Predictors for WMLs and nomogram construction

**Figure 2:**
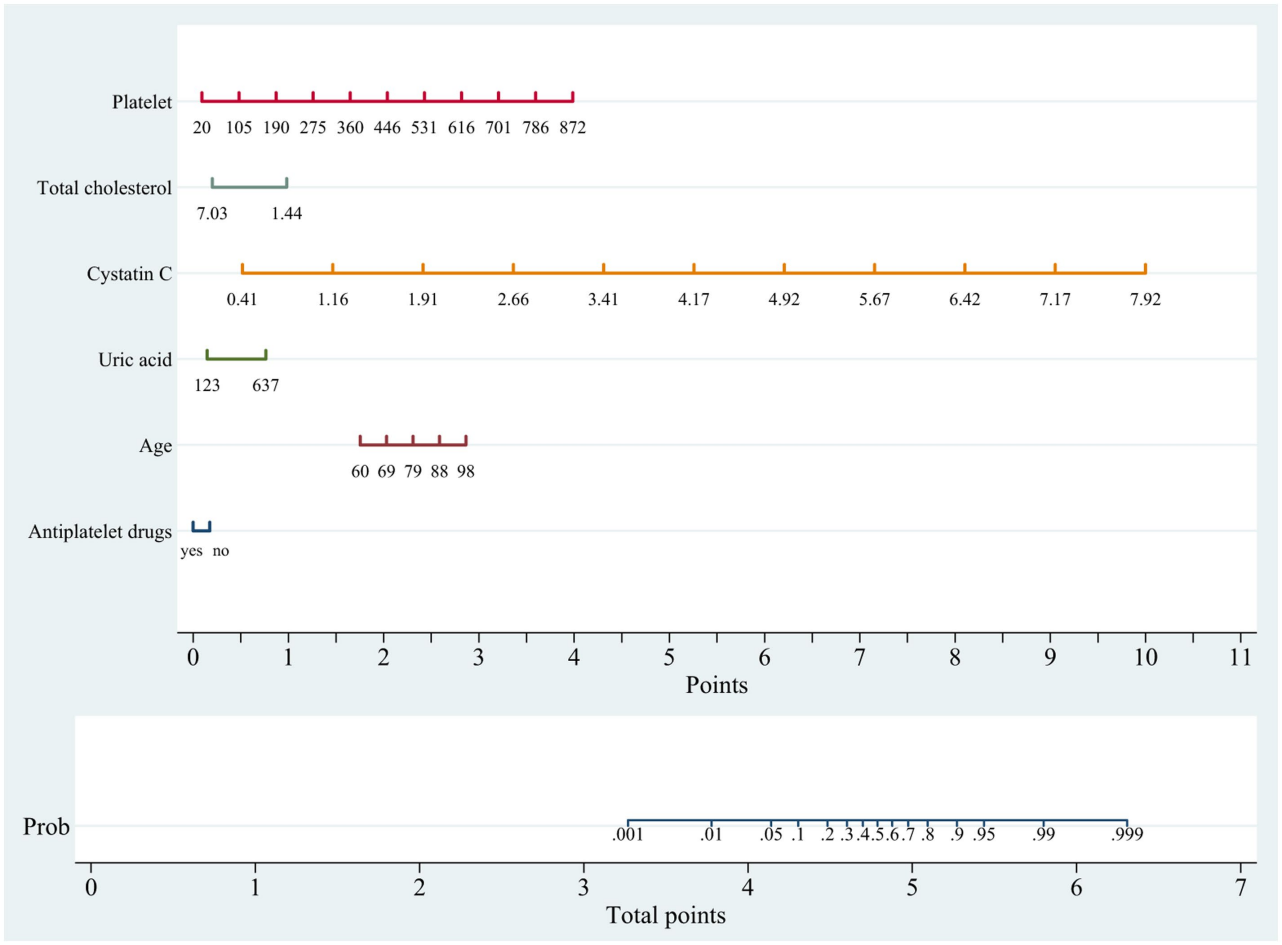
Nomogram for predicting cerebral white matter lesions in training cohort. Points are assigned based on age, cystatin C, total cholesterol, platelet, uric acid and antiplatelet drugs by drawing a line upward from the corresponding values to the “Points” line. The sum of these six points, plotted on the “Total points” line, corresponds to prediction of cerebral white matter lesions.

In the training cohort, a univariate analysis was conducted to investigate the association of several variables with WMLs. These variables include age, medical comorbidities (hypertension, CHD, tumor), SBP, use of statins, use of antiplatelet drugs, laboratory tests (Hcy, Cys C, UA, CREA, UREA, h-CRP, TC, TG, HDL, LDL, TP, ALB, BNP, HbA1C, PLT; Table 2). Finally, six variables remained significant in the multivariate logistic model, including age, cystatin C, total cholesterol, platelet, uric acid and use of antiplatelet drugs (Table 2). The predictive nomogram for WMLs that integrated these six variables was constructed and depicted in Figure 2. Based on this nomogram, the probability of predicting WMLs in patients can be calculated as follows: P_WMLs_ = 1/(1 + e^x^), x = −(−15.29 + 0.099 × age − 0.174 × antiplatelet drugs (yes) + 0 × antiplatelet drugs (no) + 6.455 × cystatin C − 0.611 × total cholesterol +0.023 × platelet +0.006 × uric acid).

**Table 2:** Univariate and multivariable logistic regression analysis of the variables associated with white matter lesions in the training cohort.

Variables	Univariate analysis		Multivariable analysis	
	OR (95% CI)	*p*	OR (95% CI)	*p*
Age	1.142 (1.11–1.18)	<0.001	1.11 (1.06–1.15)	<0.001
Weight	0.986 (0.97–1.01)	0.211	-	-
Coronary heart disease	0.318 (0.2–0.51)	<0.001	-	-
Hypertension	0.378 (0.24–0.59)	<0.001	-	-
Diabetes mellitus	0.657 (0.41–1.05)	0.077	-	-
Atrial fibrillation	0.426 (0.19–0.98)	0.044	-	-
Tumor	0.231 (0.12–0.46)	<0.001	-	-
Left heart failure	0	0.997	-	-
Smoking	0.744 (0.47–1.18)	0.209	-	-
Drinking	1.283 (0.84–1.97)	0.254	-	-
Statins	0.466 (0.29–0.74)	0.001	-	-
Antiplatelet drugs	2.12 (1.37–3.27)	0.001	0.34 (0.17–0.70)	0.003
Anticoagulants	0.51 (0.22–1.18)	0.116	-	-
Systolic blood pressure	1.035 (1.02–1.05)	<0.001	-	-
Diastolic blood pressure	0.99 (0.97–1.01)	0.388	-	-
Homocysteine	1.361 (1.24–1.49)	<0.001	-	-
Cystatin C	15545.91 (1918.72–125956.33)	<0.001	636.06 (55.07–7346.72)	<0.001
Uric acid	1.007 (1–1.01)	<0.001	1.006 (1–1.01)	0.025
Creatinine	1.045 (1.03–1.06)	<0.001	-	-
Urea nitrogen	1.369 (1.19–1.58)	<0.001	-	-
h-CRP	1.16 (1.03–1.31)	0.019	-	-
Total bilirubin	0.989 (0.95–1.03)	0.63	-	-
Direct bilirubin	1.07 (0.95–1.2)	0.253	-	-
Alanine aminotransferase	0.99 (0.97–1.01)	0.296	-	-
GGT	1.003 (0.99–1.01)	0.518	-	-
Total cholesterol	0.47 (0.37–0.59)	<0.001	0.54 (0.37–0.80)	0.002
Triglyceride	0.765 (0.59–0.99)	0.049	-	-
High density lipoprotein	0.251 (0.13–0.51)	<0.001	-	-
Low density lipoprotein	0.362 (0.26–0.5)	<0.001	-	-
Lipoprotein-A	1 (0.99–1.01)	0.823	-	-
Total protein	0.921 (0.89–0.96)	<0.001	-	-
Albumin	0.818 (0.77–0.87)	<0.001	-	-
Brain natriuretic peptide	1.006 (1–1.01)	<0.001	-	-
Glycosylated hemoglobin	1.913 (1.33–2.76)	0.001	-	-
Platelet	1.011 (1.01–1.02)	<0.001	1.02 (1.01–1.03)	<0.001

OR, odds ratio; CI, confidence interval; h-CRP, high-sensitivity C-reactive protein; GGT, gamma glutamyl transpeptidase. Results of the univariate and multivariable logistic regression model for cerebral white matter lesions using variable selection based on *p*-value significance of backward stepwise regression.

### Assessment, validation of predictive accuracy of the nomogram for WMLs

**Figure 3:**
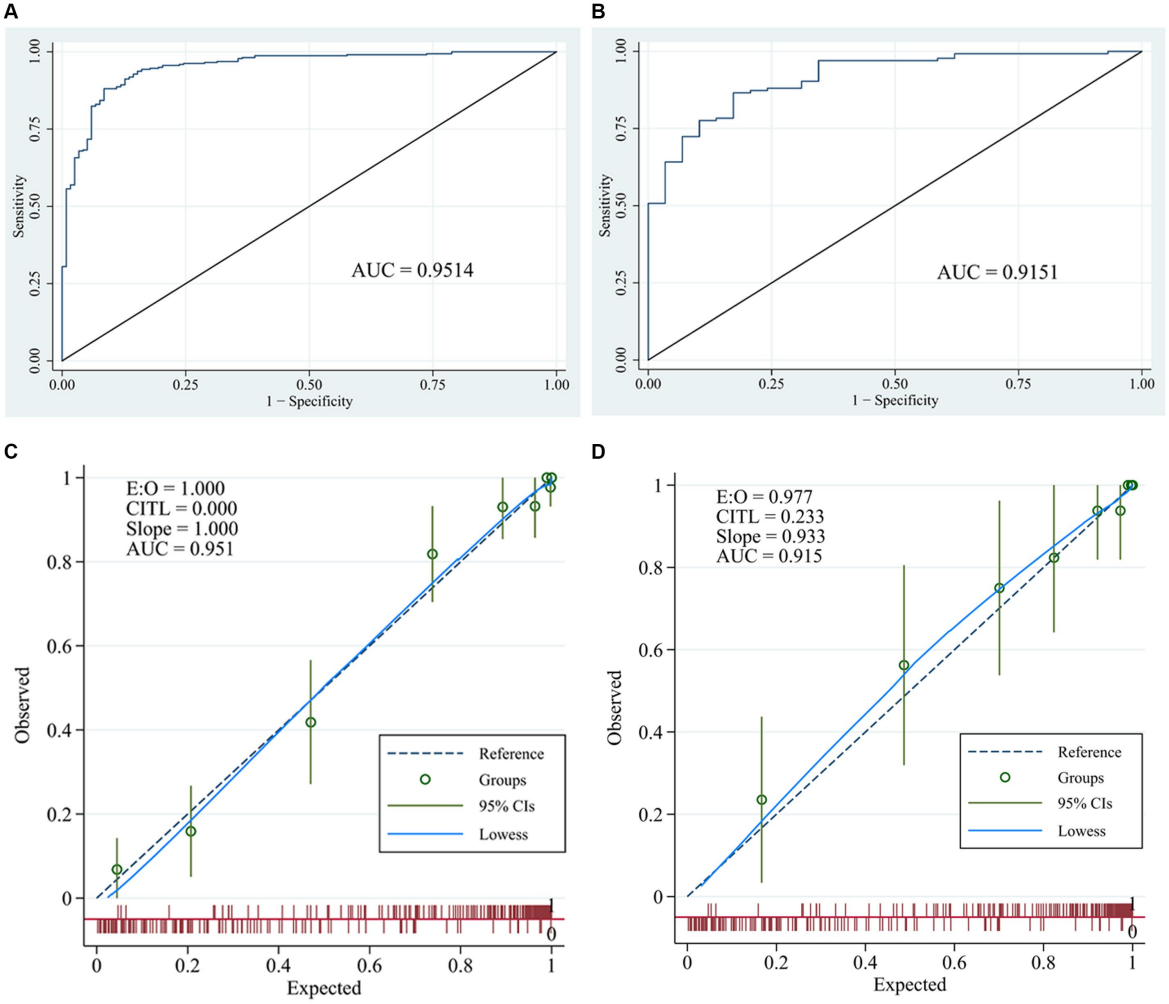
Receiver operating characteristic curves and calibration curves. ROC curve and area under the curve (AUC) in training cohort **(A)** and validation cohort **(B)** of the nomogram; calibration curve of training cohort **(C)** and validation cohort **(D)** of the nomogram.

**Figure 4:**
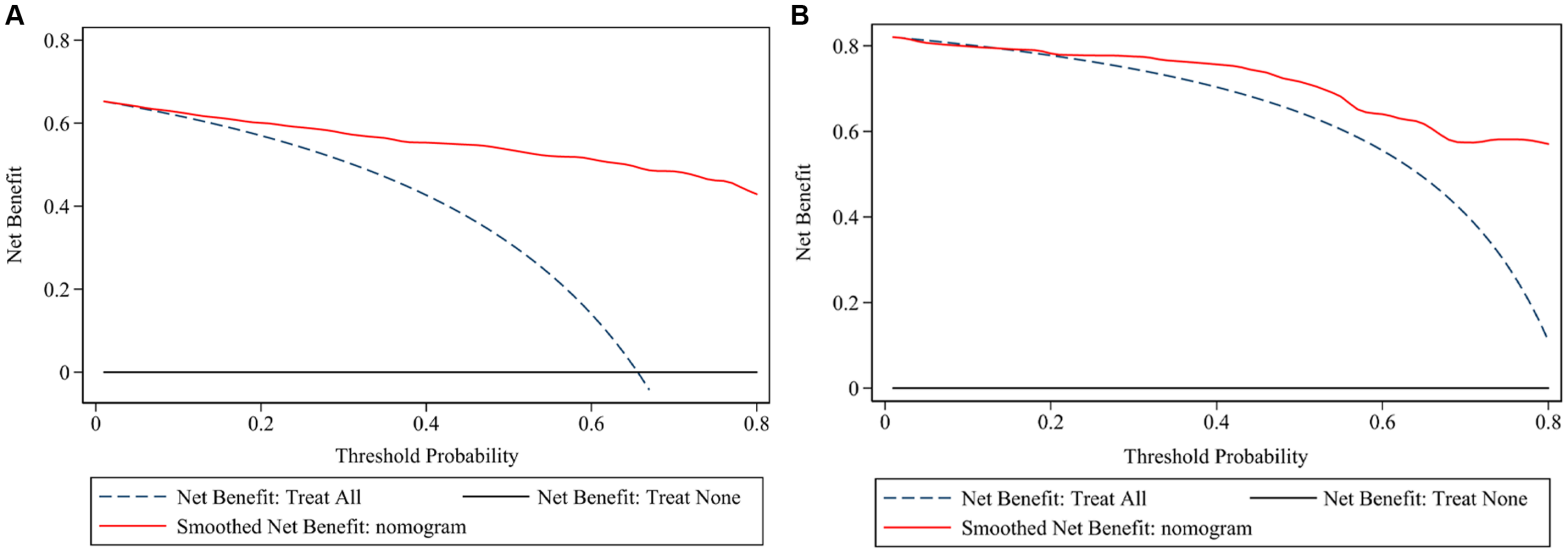
Decision curve analysis of the risk prediction model of white matter lesions. Decision curve of training cohort **(A)** and validation cohort **(B)**; the net benefit was plotted vs. the threshold probability. The horizontal black dash line depicts net benefit of a strategy of treating no patients. The blue dash line depicts net benefit of a strategy of treating all patients. The red solid line represents the nomogram.

The ROC (Figures 3A,B) and calibration curve were plotted (Figures 3C,D) to verify the accuracy and discrimination of this model. The AUC in the training cohort was 0.951 (95% CI, 0.929–0.972), while 0.915 (95% CI, 0.864–0.966) in the validation cohort, which suggested that our model had good accuracy. The calibration curve of the training cohort and validation cohort showed that there was excellent consistency between the predicted survival probability and the actual survival probability. The Hosmer-Lemeshow test for the training cohort yielded a *p*-value of 0.594, suggesting that the nomogram had a perfect fit. The calibration of the nomogram was further confirmed with the validation cohort, for which the Hosmer-Lemeshow test yielded a *p*-value of 0.178, suggesting no departure from the good fit of the nomogram. DCA was also plotted to evaluate the usefulness of the nomogram in the clinical utility (Figures 4A,B) and the results showed that the predictive nomogram provided sound clinical guidance with a good net benefit.

## Discussion

In the current study, we developed and validated a predictive nomogram to identify the increased risk of WMLs in the male elderly patients over 60 years old. The newly developed model incorporated several key factors, including age, TC, PLT, Cys C, UA, and use of antiplatelet drugs. The resulting nomogram showed excellent predictive power as well as good discrimination and calibration.

Among these six variables, age, Cys C, and platelet were found to have a greater impact on WMLs. It was expected that age was found as a main contributor of this model, because age has been well demonstrated to be associated with WMLs (13–15). Given the inhibitory effect of increased serum Cys C on the contractile function of arterioles (16), and the damaging effect on the blood–brain barrier (17), the close association of Cys C with WMLs was explainable in the current study. In agreement with prior studies, platelet was found to be associated with WMLs in this study. Kuriyama et al. (18) found that the risk of WMLs in the elderly with high platelet activity increased by about threefold. Higher platelet aggregation rate have been linked to a higher incidence of WMLs, with a risk significantly higher than classical vascular risk factors such as hypertension, diabetes, and age (19). In addition, aspirin administration in patients with WMLs not only prevent cerebral infarction, but also curb the progress of WMLs (20), which was in agreement with the current finding that antiplatelet drug was independently associated with WMLs. Collectively, these results suggest the potential association of platelet and use of antiplatelet drug with WMLs.

In the current study, UA was also found to be associated with WMLs. Prior studies have found that hyperuricemia was an independent risk factor for WMLs (21). The underlying mechanisms included its effect on vascular endothelial cell dysfunction and atherosclerosis (22). Remarkably, our study found that lower TC levels may lead to an increased risk of WMLs, which seems contradictory with previous studies reporting that high cholesterol levels increased the risk of WMLs (23, 24). We argue that there are two possible reasons. First, as the main components of cell membrane and nerve myelin sheath, cholesterol plays a fundamental role in the development of the central nervous system and the creation and maintenance of new synapses (25, 26). This could explain the effect of significantly lower cholesterol levels on chronic cerebral injury, such as the developments of WMLs. Second, many studies have confirmed that intensive lipid reduction will greatly increase the risk of cerebral micro bleeds (CMBs) (27, 28), while there was a close correlation between CMBs and the formation of WMLs (29), which may explain the association of significantly lower cholesterol levels with WMLs. Furthermore, the current finding was supported by some studies reporting that the increase of TC was considered to be a protective factor of WMLs (30, 31). In addition, some studies did not find a correlation between TC and WMLs (32). Although there was a higher prevalence of diabetes and hypertension in the current study, we did not find the association of diabetes, hypertension and blood pressure with WMLs, which were known to be associated with WMLs (33–35). One possible explanation is that the blood pressure and blood glucose were well controlled in this cohort (36, 37). In addition, high prevalence of WMLs in both cohorts due to elderly patients (older than 60) and high prevalence (>60%) of hypertension may mask their associations, so their associations with severity of WMLs deserve to be further investigated. However, some studies did not find a correlation between diabetes and WMLs (38, 39), which was in line with our findings.

Our study possesses two potential strengths. First, our nomogram was created based on analysis of comprehensive and routinely available baseline variables in a training cohort, which was further validated in a follow-up cohort. Second, Cys C, UA, TC, platelet, and the use of antiplatelet drugs were incorporated into the prediction model for the first time and had excellent predictive power, and good discrimination and calibration. Nevertheless, we acknowledge several limitations. First, this was a retrospective observational study, which had common limitations including bias, potential lack of baseline data and inevitable confounding effect, although baseline data were comprehensively collected. Second, this was a single-center study without an external validation cohort, but it was validated by a follow-up cohort. Third, as almost all the patients in our center were male, our study did not include female patients. So, this finding is only applicable to male patients. Fourth, we did not collect the data about other brain lesions or underlying disease, which might have effect on this finding given their associations with WMLs. Finally, further confirmation of these findings in non-Chinese populations would be needed, given the differences in body mass and comorbid factors compared with other populations.

## Conclusion

A novel nomogram tool has been developed to predict WMLs with excellent predicting power, good discrimination and calibration. The model should be helpful to identify elderly men at high risk of cerebral WMLs.

## Data availability statement

The raw data supporting the conclusions of this article will be made available by the authors, without undue reservation.

## Ethics statement

The studies involving humans were approved by the Institutional Review Board of General Hospital of Northern Theater Command. The studies were conducted in accordance with the local legislation and institutional requirements. The ethics committee/institutional review board waived the requirement of written informed consent for participation from the participants or the participants’ legal guardians/next of kin because this is a retrospective study.

## Author contributions

Y-FP: Data curation, Formal analysis, Writing – original draft. X-DL: Data curation, Formal analysis, Writing – original draft. Q-YL: Data curation, Formal analysis, Writing – original draft. C-WZ: Formal analysis, Writing – original draft. Y-HW: Data curation, Writing – original draft. M-RC: Writing – original draft. H-SC: Funding acquisition, Supervision, Writing – review & editing.
